# Visualization of Intrapulmonary Lymph Vessels in Healthy and Inflamed Murine Lung Using CD90/Thy-1 as a Marker

**DOI:** 10.1371/journal.pone.0055201

**Published:** 2013-02-08

**Authors:** Sarah Kretschmer, Ina Dethlefsen, Stefanie Hagner-Benes, Leigh M. Marsh, Holger Garn, Peter König

**Affiliations:** 1 Institut für Anatomie, Zentrum für medizinische Struktur- und Zellbiologie, Universität zu Lübeck, Lübeck, Germany; 2 Institut für Laboratoriumsmedizin und Pathobiochemie, Molekulare Diagnostik, Philipps-Universität, Marburg, Germany; 3 Ludwig Boltzmann Institute for Lung Vascular Research, Graz, Austria; McGill University, Canada

## Abstract

**Background:**

Lymphatic vessels play a pivotal role in fluid drainage and egress of immune cells from the lung. However, examining murine lung lymphatics is hampered by the expression of classical lymph endothelial markers on other cell types, which hinders the unambiguous identification of lymphatics. The expression of CD90/Thy-1 on lymph endothelium was recently described and we therefore examined its suitability to identify murine pulmonary lymph vessels under healthy and inflammatory conditions.

**Methodology/Principal Findings:**

Immunohistochemistry with a monoclonal antibody against CD90.2/Thy-1.2 on 200 µm thick precision cut lung slices labeled a vascular network that was distinct from blood vessels. Preembedding immunostaining and electron microscopy verified that the anti-CD90.2/Thy-1.2 antibody labeled lymphatic endothelium. Absence of staining in CD90.1/Thy-1.1 expressing FVB mice indicated that CD90/Thy-1 was expressed on lymph endothelium and labeling was not due to antibody cross reactivity. Double-labeling immunohistochemistry for CD90/Thy-1 and α-smooth muscle actin identified two routes for lymph vessel exit from the murine lung. One started in the parenchyma or around veins and left via venous blood vessels. The other began in the space around airways or in the space between airways and pulmonary arteries and left via the main bronchi. As expected from the pulmonary distribution of lymph vessels, intranasal application of house dust mite led to accumulation of T cells around veins and in the connective tissue between airways and pulmonary arteries. Surprisingly, increased numbers of T cells were also detected around intraacinar arteries that lack lymph vessels. This arterial T cell sheath extended to the pulmonary arteries where lymph vessels were located.

**Conclusions/Significance:**

These results indicate that CD90/Thy-1 is expressed on lymphatic endothelial cells and represents a suitable marker for murine lung lymph vessels. Combining CD90/Thy-1 labeling with precision cut lung slices allows visualizing the anatomy of the lymphatic system in normal and inflamed conditions.

## Introduction

Lymph vessels are important to drain excess extracellular fluid [1] and transport antigens as well as immune cells to lymph nodes to initiate an adaptive immune reaction [2]. Especially in the lung, fluid balance and efficient immune reactions are essential since accumulation of fluid as well as failure to fight infections may lead to impairment of gas exchange and ultimately death. Despite the fact that a functioning lymphatic system is essential for lung function, research on the lymphatic system in the respiratory system in mice largely focussed on the trachea [3], [4], [5], [6]. This is mostly due to the fact that classical lymph vessel markers such as podoplanin or LYVE-1, which are widely used to identify lymph vessels in other organs also label other cell types in the lung that hamper the unequivocal identification of lymph vessels [7], [8].

Recently, CD90/Thy-1 was found to be highly expressed on lymphatic endothelial cells and was implied to facilitate cell entry into lymphatic vessels [9]. CD90/Thy-1 is a glycophosphatidylinositol anchored strongly glycosylated protein that is expressed on the cell surface and belongs to the class of the immunoglobulin superfamily CD90/Thy-1 was originally identified as a thymocyte antigen and is a pan T cell marker in mice [10]. It is also known to be expressed by neurons [11] and fibroblasts [12].

The goal of this study was to clarify if CD90/Thy-1 is also expressed on lung lymphatic endothelial cells and could serve as a valuable marker for lymphatics in the lung under normal and inflammatory conditions.

Our results show that CD90/Thy-1 is expressed strongly on lymph vessel endothelium in the murine lung and can be used to reliably identify lymph vessels in murine precision cut lung slices even under inflammatory conditions. By combining labeling for CD90/Thy-1 with precision cut lung slices we could identify the anatomy of the intrapulmonary lymphatic vasculature and gain insight into possible fluid drainage and immune cell routes in the murine lung.

## Materials and Methods

### Mice

For light and electron microscopic studies of normal lungs, male C57BL/6 mice (Charles River Laboratories Research Models and Services Germany GmbH, Sulzfeld, Germany) aged 8 to 30 weeks were used. For the model of allergic airway inflammation female Balb/c mice (Charles River) aged 8 weeks were used. Both, C57BL/6 and Balb/c mice express the CD90.2 variant of CD90/Thy-1. To control for the specificity of the anti-CD90.2/Thy-1.2 antibody FVB mice (Charles River) were used that express the CD90.1 variant of CD90/Thy-1. All animals were held according to institutional guidelines with a 12 h day/night cycle and food and drink ad libitum. All animal experiments were approved by the Ministerium für Landwirtschaft, Umwelt und ländliche Räume des Landes Schleswig-Holstein or the Regierungspräsidium Giessen and measures were taken to keep animal suffering to a minimum.

### Antibodies Used

Primary antibodies and concentrations used for light microscopy: rat anti-CD90.2/Thy-1 antibody (clone 30-H12, BD Biosciences Pharmingen, Heidelberg, Germany, 1∶3000), anti-CD90.2/Thy-1 antibody conjugated with Alexa Fluor 488 (clone 30-H12, BioLegend Europe BV, Uithoorn, Netherlands, 1∶1000); rat anti-CD90.2/Thy-1 antibody conjugated with FITC (clone 30-H12, Leinco Technologies Inc., St. Louis, USA, 1∶2000), rat anti-CD90.1/Thy-1 antibody conjugated with Alexa Fluor 488 (clone OX-7, BioLegend Europe, 1∶2000); syrian hamster anti-podoplanin antibody (clone 8.1.1, BioLegend Europe, 1∶600); rat anti-CD45 antibody, biotinylated, (clone 30-F11, BD Biosciences, Heidelberg, Germany, 1∶1000), armenian hamster anti-CD3e antibody (clone 145-2C11, BD Bioscience Pharmingen, 1∶200), rabbit anti-LYVE-1 antibody (polyclonal, Acris Antibodies GmbH, Herford, Germany, 1∶1000), rabbit anti-LYVE-1 antibody (polyclonal, AngioBio, Del Mar, Ca, USA, 1∶3000); mouse anti-α-smooth muscle actin antibody conjugated with Cy3 (clone 1A4, Sigma Aldrich, Seelze, Germany, 1∶100), goat anti-mouse VEGF R3 antibody (polyclonal R&D Systems Europe, Ltd., Abingdon, United Kingdom, 1∶150).

Secondary reagents and concentrations used: donkey anti-rabbit immunoglobulin (Ig) antibody conjugated with Alexa Fluor 488 (1∶800), donkey anti-rabbit Ig antibody conjugated with Alexa Fluor 555 (1∶500), donkey anti-rat Ig antibody conjugated with Alexa Fluor 488 (1∶400), donkey anti-rat Ig antibody conjugated with Alexa Fluor 555 (1∶3000), goat anti-syrian hamster Ig antibody conjugated with Alexa Fluor 568 (1∶200), donkey anti-goat Ig antibody conjugated with Alexa Fluor 555 (1∶100), streptavidin conjugated with Alexa Fluor 555 (1∶1600), all from Invitrogen and donkey anti-armenian hamster Ig antibody conjugated with Dylight 549 (1∶200) from Jackson ImmunoResearch (Newmarket, Suffolk, England).

### Precision Cut Lung Slices

Animals were sacrificed by an overdose of isoflurane (Baxter, Unterschleißheim, Germany). The thorax was opened and the trachea was exposed. Blood from the lung was removed by cutting the left auricle and injecting 5 ml of HEPES-buffered Ringer solution into the right ventricle. The heart was then removed and the lung was filled via the trachea with 37°C warm 3% low melting point agarose (Bio Rad, München, Germany) in HEPES-Ringer solution. The lung was removed from the thorax en bloc and transferred to ice cold HEPES-buffered Ringer solution. The left lung was isolated and was glued with the hilum facing downwards on cooled aluminium block using super glue (UHU, Bühl, Germany). Then, 200 µm thick sections were cut using a vibratome. The individual sections were collected and were subsequently incubated in 37°C warm HEPES-buffered Ringer solution with continuous bubbling for 3 h with room air to remove the agarose. Sections were fixed in 4% paraformaldehyde (PFA) in phosphate buffered saline (PBS, pH 7.4) and washed three times. After incubation overnight in 20% sucrose the sections were frozen in the same solution at −20°C until further use.

### Fluorescence Immunohistochemistry for Light Microscopy

Cryosections (10 µm thickness) from 4% paraformaldehyde fixed and sucrose cryoprotected lungs or precision cut lung slices were incubated with primary antibodies diluted in TRIS-buffered saline (TBS, pH 7.4) overnight. If fluorophore-conjugated primary antibodies were used, the sections were washed in TBS and coverslipped (sections on slides) or mounted between two cover slips (precision cut lung slices) using buffered Mowiol 4–88, pH 8.5 (Sigma-Aldrich). To detect non labeled or biotiylated antibodies, sections were washed in TBS and incubated with fluorophore-conjugated secondary antibodies or with fluorophore conjugated streptavidin, respectively for one hour. Then sections were rinsed in TBS and coverslipped. For double-labeling of CD45 and CD90/Thy-1 two modified protocols were used since both antibodies were raised in rat. Both gave similar results. First, sections were incubated with anti-CD90/Thy-1 antibody as described above and detected using anti-rat Ig antibody conjugated with Alexa Fluor 488. Then, sections were incubated with normal rat serum and fixed with 4% paraformaldehyde in PBS to block potentially free binding sites of the secondary antibody. After a subsequent rinsing step, the slices were incubated with the biotinylated rat anti-CD45 antibody overnight and detected using streptavidin coupled to Alexa Fluor 555. Alternatively, the sections were incubated with biotinylated anti-CD45 antibody overnight and detected using a donkey anti-rat Ig antibody coupled to Alexa Fluor 555. After washing, sections were incubated with normal rat serum and fixed with 4% paraformaldehyde to block free binding sites of the secondary antibody. Subsequently, the sections were incubated with anti-CD90/Thy-1 antibody coupled to Alexa Fluor 488 overnight, rinsed and coverslipped. Sections were evaluated by using an epifluorescence microscope (Axioskop 2 FS plus, Carl Zeiss Microscopy GmbH, Jena, Germany) with appropriate filter sets or a confocal laser scanning microscope (510 Meta, Carl Zeiss Microscopy GmbH).

### Preembedding Immunohistochemistry for Electron Microscopy

For these experiments lungs from six C57BL/6 mice were used. After thawing, the precision cut lung slices were transferred to anti-CD90/Thy-1 antibody (1∶3000 in antibody diluent (Invitrogen) or PBS) and incubated overnight at 4°C. Subsequently, sections were washed in PBS and incubated with biotinylated anti-rat Ig antibody (Dako, Hamburg, Germany, 1∶400) with 5% normal mouse serum (Invitrogen) in antibody diluent for 1 h. After a washing step, sections were incubated with Vectastain Elite ABC reagent (Elite PK 6100 Standard, Vectorstain Linaris Biologische Produkte GmbH, Wertheim-Bettingen/Vector Laboratories, Burlingame, CA) for 45 min. Diaminobenzidine solution (Vector Laboratories) was added and sections were incubated under microscopic control until sufficient immunostaining was achieved. Sections were then washed in PBS and fixed in Monti’s fixative (2% glutardialdehyde, 0.6% paraformaldehyde, and 0.03% CaCl2 in 0.06 M cacodylate buffer, pH 7.35 [13]) overnight and transferred to in 0.1 M cacodylate buffer, pH 7.3, followed by incubation with 1% osmiumtetroxide in cacodylate buffer overnight. After washing in cacodylate buffer, sections were dehydrated by incubating them in increasing concentrations of ethanol, transferred to propylene oxide, and left in a 1∶1 mixture of Araldite (Sigma-Aldrich) and propylenoxide overnight and were transferred to Araldite for 30 min and then to fresh Araldite. The sections were flattened between two glass slides covered by polyethylene foil and were left at 60°C for two days. After polymerization the foil was removed and sections were evaluated under a microscope. Suitable areas were cut and glued to an Araldite resin blind block. Semi thin sections were cut using an ultramicrotome (Ultracut E, Leica Microsystems, Wetzlar, Germany). If a section contained a stained lymph vessel, ultrathin sections were cut at approx. 80 nm and were transferred to copper grids. Sections were contrasted in a contrasting system for ultrathin sections (Leica EM AC20, Leica Microsystems) using uranyle acetate ready to use solution (Leica Microsystems) followed by lead citrate ready to use solution (Leica Microsystems) and were subsequently evaluated using a transmission electron microscope (Jeol JEM 1011, Eching, Germany).

### Paraffin Sections

Lungs from C57BL/6 mice were fixed via the trachea with 4% PBS-buffered PFA (pH 7.4) and embedded in paraffin. Paraffin sections were cut and stained with Masson Goldner trichrome stain (Sigma-Aldrich). Sections then were coverslipped and evaluated by light microscopy.

### Multiphoton Microscopy

Murine tracheae from C57BL/6 were explanted and kept at 37°C in HEPES Ringer solution throughout incubation and imaging. FITC-conjugated rat anti-CD90/Thy-1 antibody was added to the HEPES Ringer solution. After 1 h the tissue was transferred to a DermaInspect multiphoton microscope (JenLab GmbH, Jena, Germany) equipped with a 20× dip in objective, (NA = 1.0, Carl Zeiss Microscopy GmbH) and a Mai Tai mode-locked titanium-sapphire laser (Newport Spectra-Physics GmbH, Darmstadt, Germany). Z-stacks were recorded using an excitation wavelength of 750 nm and detecting autofluorescence and FITC signals.

### Model of Allergic Airway Inflammation

Female Balb/c mice (n = 6) were treated intranasally with a crude extract of house dust mite (Greer, Lenoir, NC, USA) in PBS (100 µg/50 µl) or PBS alone (n = 6) on days 0, 7, and 14. On Day 15 precision cut lung slices from the left lungs were prepared and immunostained for light microscopic analysis with anti-CD90/Thy-1 antibody and anti-α-smooth muscle actin antibody as described above.

## Results

### Morphology of CD90/Thy-1-immunoreactive Vessels

**Figure 1:**
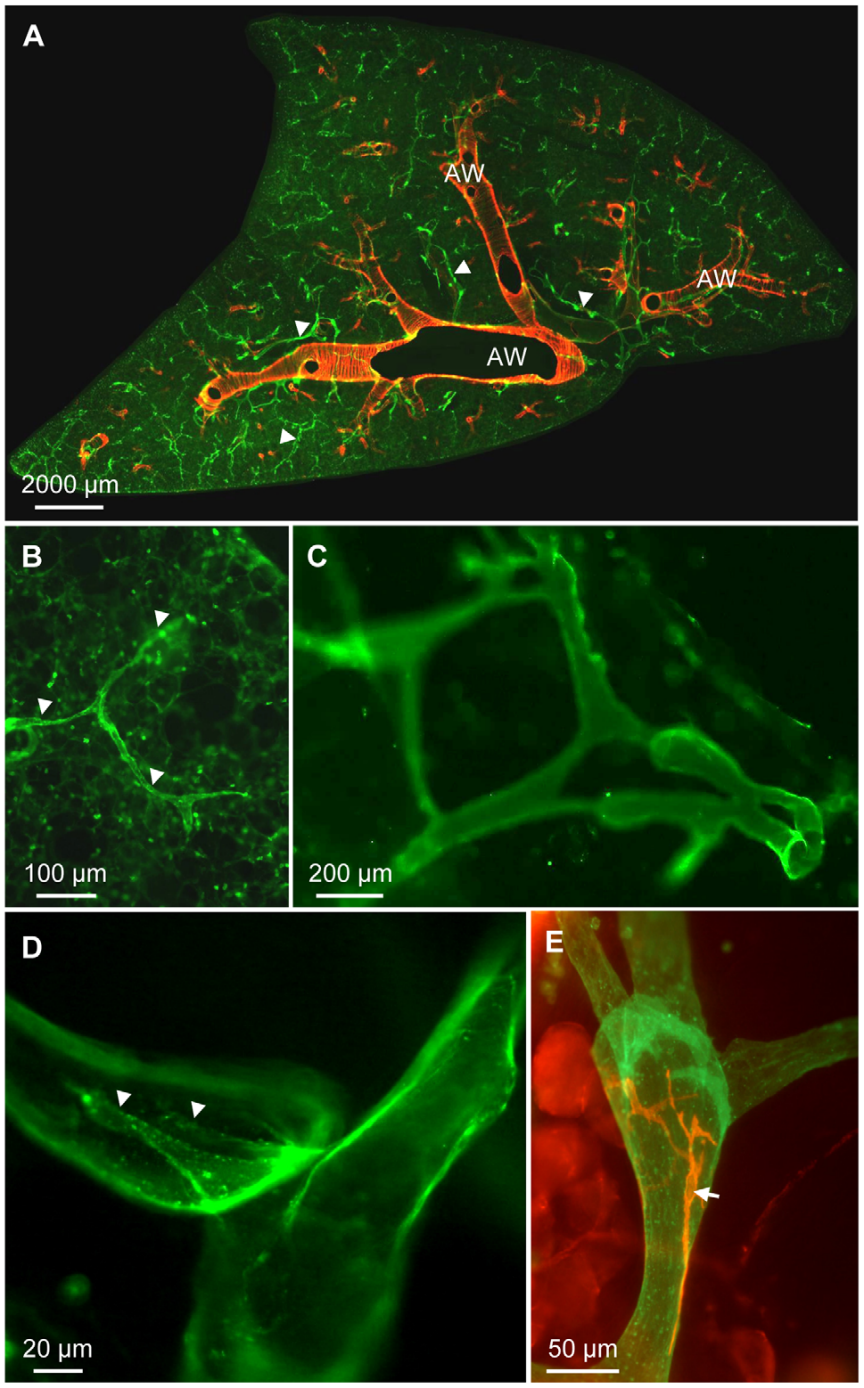
Immunohistochemistry of CD90/Thy-1 in murine precision cut lung slices. A) The anti-CD90/Thy-1 antibody (green) stains a vascular system (arrowheads) in murine precision cut lung slices that is distributed throughout the lung. Red staining shows immunoreactivity for α-smooth muscle actin. AW: airways. B) Initial CD90/Thy-1-immunoreactive capillaries (arrowheads) in the alveolar region. C) The CD90/Thy-1-immunoreactive vascular network is interconnected. D) A CD90/Thy-1-immunoreactive valve (arrowheads). E) Only close to the hilum, α-smooth muscle actin-immunoreactive cells (red, arrow) were found on CD90/Thy-1-immuoreactive vessels.

In precision cut lung slices the anti-CD90/Thy-1 antibody strongly labeled vessels that were found throughout the murine lung (Figure 1A). The vessels started as blind capillaries in the parenchyma (Figure 1B), were interconnected and exhibited variable changes in diameter (Figure 1C). Larger vessels occasionally contained valve like structures (Figure 1D). With the exception of large vessels close to the hilum (Figure 1E), CD90/Thy-1-immunoreactive vessels were not surrounded by smooth muscle cells as judged by absence of α-smooth muscle actin positive cells. The analysis is based on n = 9 animals.

To verify that the CD90/Thy-1-immunoreactive cells were lymph vessels, we used preembedding immunohistochemistry on precision cut lung slices.

**Figure 2:**
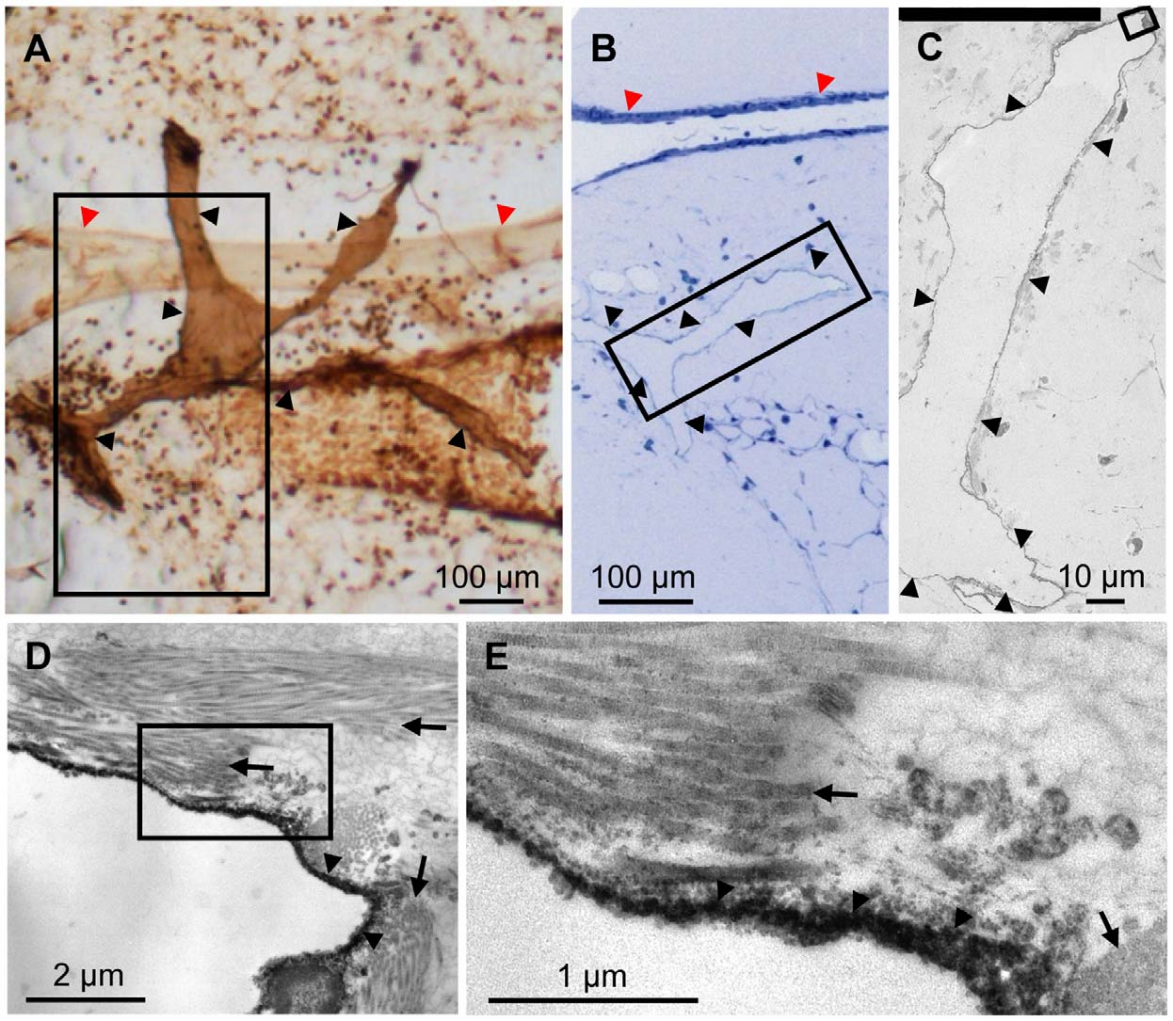
Preembedding staining for CD90/Thy-1 in murine precision cut lung slices. A) Intense immunostaining for CD90/Thy-1 is found in vascular structures. B) Boxed area in A, is shown as semi thin section. C) Boxed area in B, is shown as low magnification electron micrograph. D) Boxed area in C, is shown in higher magnification showing reaction product on an endothelial cell (arrowheads) that exhibited intimate contact to collagen fibers. E) Boxed area in D. Black arrowheads in A–E: immunoreactive lymph vessel, red arrowheads in A, B: pulmonary artery, arrows in D, E: collagen fibers.

Immunoreactive vessels were identified in precision cut lung slices by their dark staining and could be readily identified in semi thin sections (Figure 2A, B). Using electron microscopy electron dense reaction product was found on endothelial cells of vessels that neither had smooth muscle cell nor pericyte coating. Labeled endothelial cells were in intimate contact to collagen fibers (Figure 2C–E). Absence of pericytes and smooth muscle cells and intimate contact to collagen fibers is a hallmark of lymph vessels. The analysis is based on n = 6 animals.

### Double-labeling of CD90/Thy-1 with Lymph Vessel Markers

**Figure 3:**
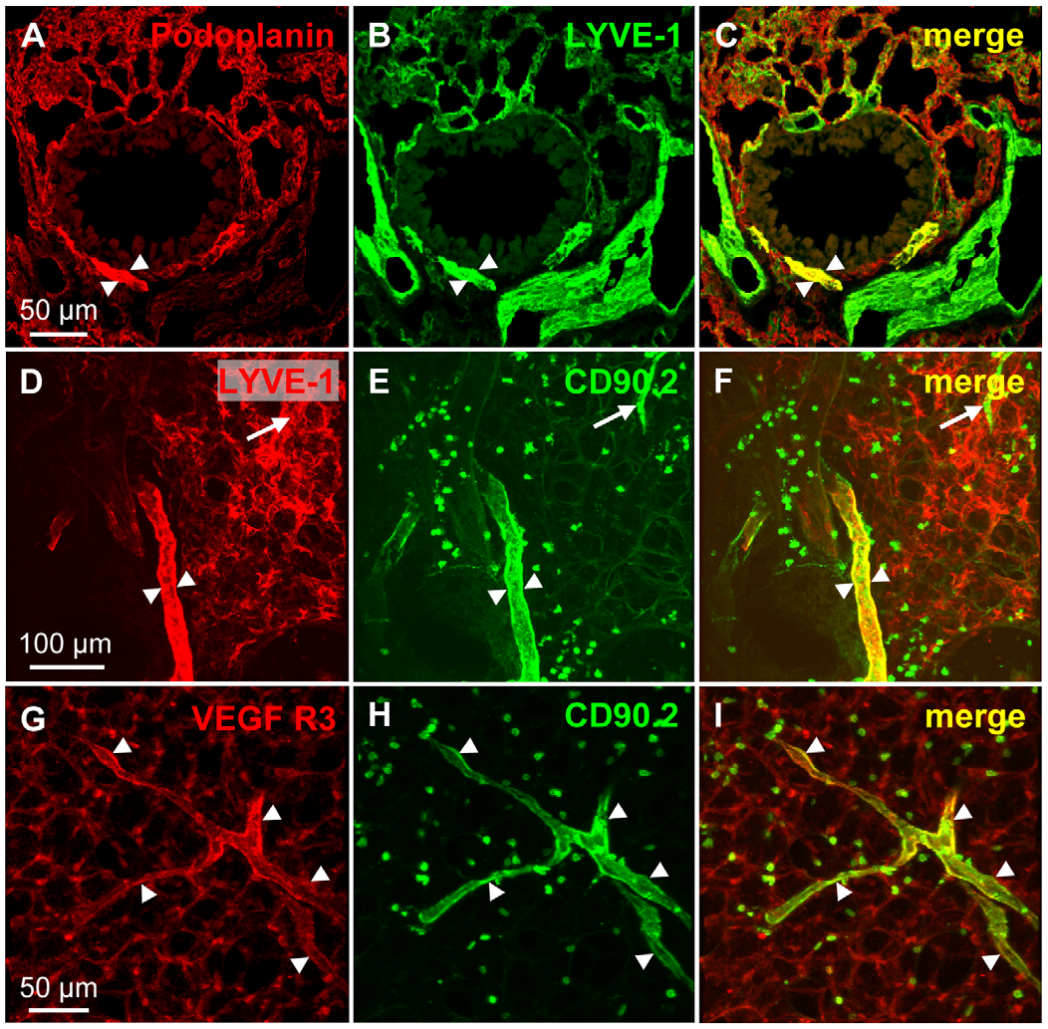
Double-labeling of CD90/Thy-1 with established lymph vessel markers. A–C) Cryostat section. Podoplanin and LYVE-1 not only label lymph vessels (arrowheads) but also other cell types in the murine lung. D–F) Precision cut lung slice. Double-labeling of LYVE-1 with CD90/Thy1 shows that a lymph vessel (arrowheads) is labeled by both antibodies but staining of other cells prevents identification of lymph vessels in the alveolar region (arrow). G–I) Precision cut lung slice. Co-labeling with antibodies against VEGF R3 and CD90/Thy-1 shows colocalization in lymph vessels in the alveolar region (arrowheads) but the antibody to VEGF R3 binds to other structures in the alveolar region.

LYVE-1 and podoplanin labeled also other cells strongly impairing their use as lymph vessel markers in the murine lung (Figure 3A–C). Double-labeling of CD90/Thy-1 with antibodies against the established lymph vessel markers LYVE-1 (Figure 3D–F), and VEGF R3 (Figure 3G–I) confirmed that the CD90/Thy-1-immunoreactive vessels are lymphatic vessels. However, also the anti-VEGF R3 antibody gave a weak staining with considerable background in the alveolar region compared to the anti-CD90/Thy-1 antibody limiting its use in precision cut lung slices. The analysis is based on n = 3−5 animals.

### Specificity of the Anti-CD90/Thy-1 Antibody

**Figure 4:**
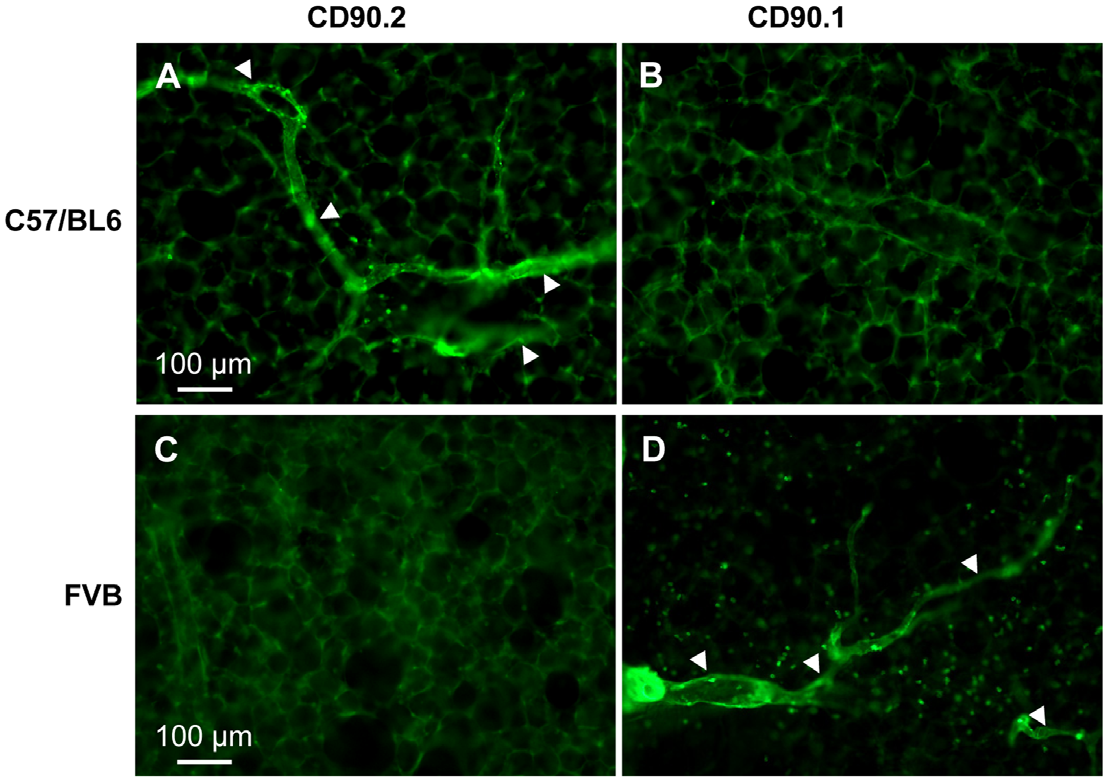
Immunostaining for CD90/Thy-1 in C57BL/6 mice and FVB mice. (A, B) The anti-CD90/Thy-1 antibody used that is directed against the CD90.2 variant labels cells in precision cut lung slices in C57BL/6 mice (A) that express CD90.2 but not in FVB mice (B) that express the CD90.1 variant. C, D) Conversely, the anti-CD90.1/Thy-1.1 antibody used, does not label lymph vessels in C57BL/6 mice (C) that express CD90.2 but labels lymph vessels in FVB mice (D) expressing CD90.1. Arrowheads = labeled lymph vessels.

To verify that lymph vessels indeed expressed CD90/Thy-1, we used FVB mice that express the CD90.1 variant of CD90/Thy-1, which is not recognized by the anti-CD90.2/Thy-1.2 antibody. In precision cut lung slices from FVB mice no staining of lymph vessels (Figure 4) or any other cells was detected with the anti-CD90.2./Thy-1.2 antibody. Conversely, use of an anti-CD90.1/Thy1.1 antibody labeled lymph vessels in FVB mice but not in C57BL/6 mice indicating that the staining of the anti-CD90/Thy-1 antibodies was specific for CD90/Thy-1 and that lymph vessels indeed express CD90/Thy-1. The analysis is based on n = 3 FVB mice and n = 3 C57BL/6 mice.

### Distribution of Lymph Vessels in the Normal Murine Lung

**Figure 5:**
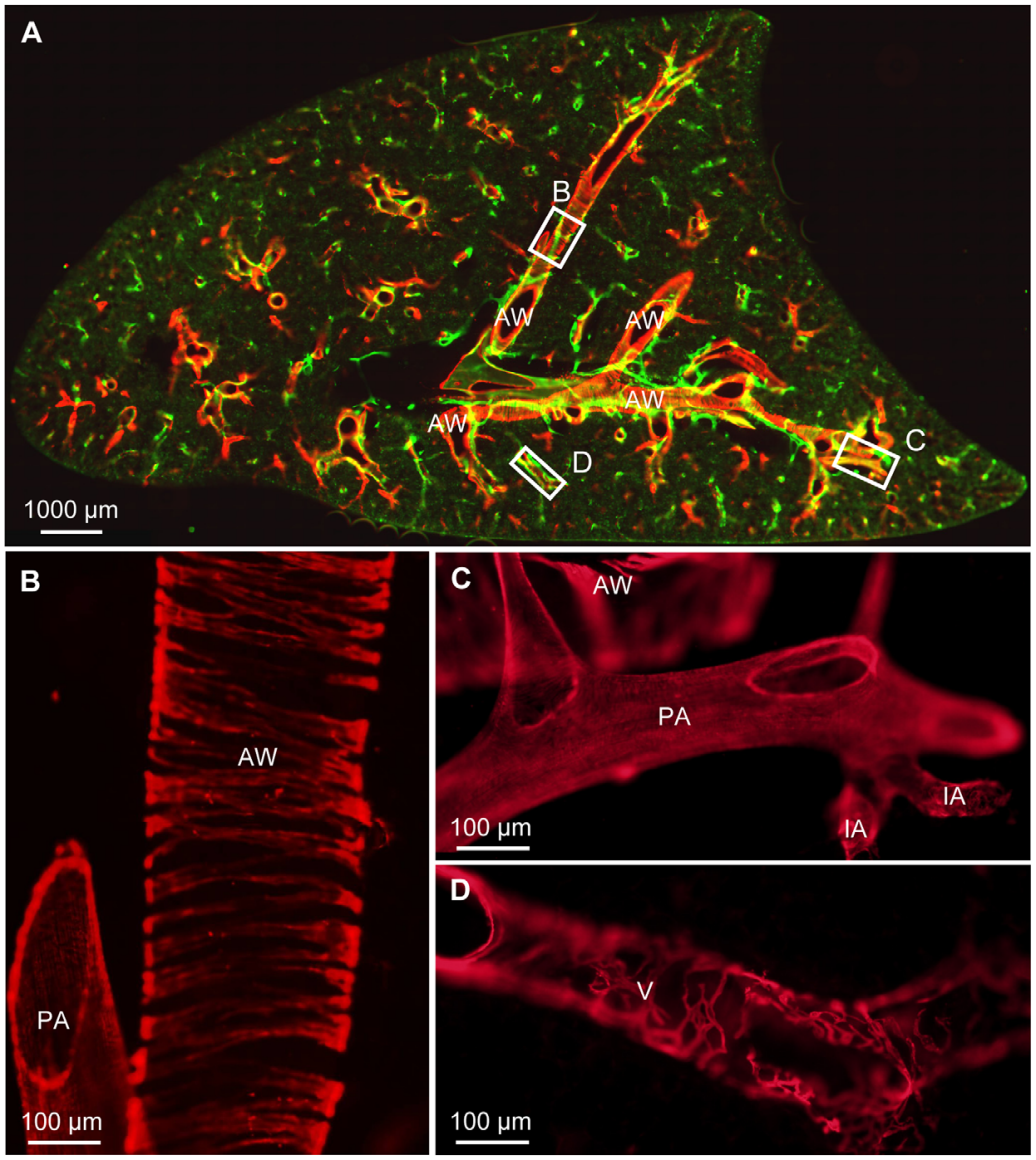
Identification of airways, arteries, and veins by α-smooth muscle actin staining in murine precision cut lung slices. A) Precision cut lung slice stained with antibodies against α-smooth muscle actin (red) and CD90/Thy-1 (green). Based on the location and their characteristic architecture of smooth muscle cells, airways, arteries, and veins can be distinguished. B) Boxed area in A labeled with B. An airway (AW) is accompanied by a pulmonary artery (PA). The airway is identified by smooth muscle cells that are oriented perpendicular to course of the airway lumen and exhibit regular gaps between them. C) Boxed area in A labeled with C. Pulmonary arteries are identified by their continuous layer of α-smooth muscle actin stained smooth muscle cells and their proximity to airways. Smaller intraacinar arteries (IA) branch from the pulmonary artery, accompany alveolar ducts (not visible here) and subsequently lose their coating of α-smooth muscle actin-immunoreactive cells. D) Boxed area in A labeled D. Pulmonary veins (V) are identified by their coating of irregularly formed α-smooth muscle actin-immunoreactive cells that form a lose mesh around the vessel. They do not accompany airways or alveolar ducts and lie separately.

**Figure 6:**
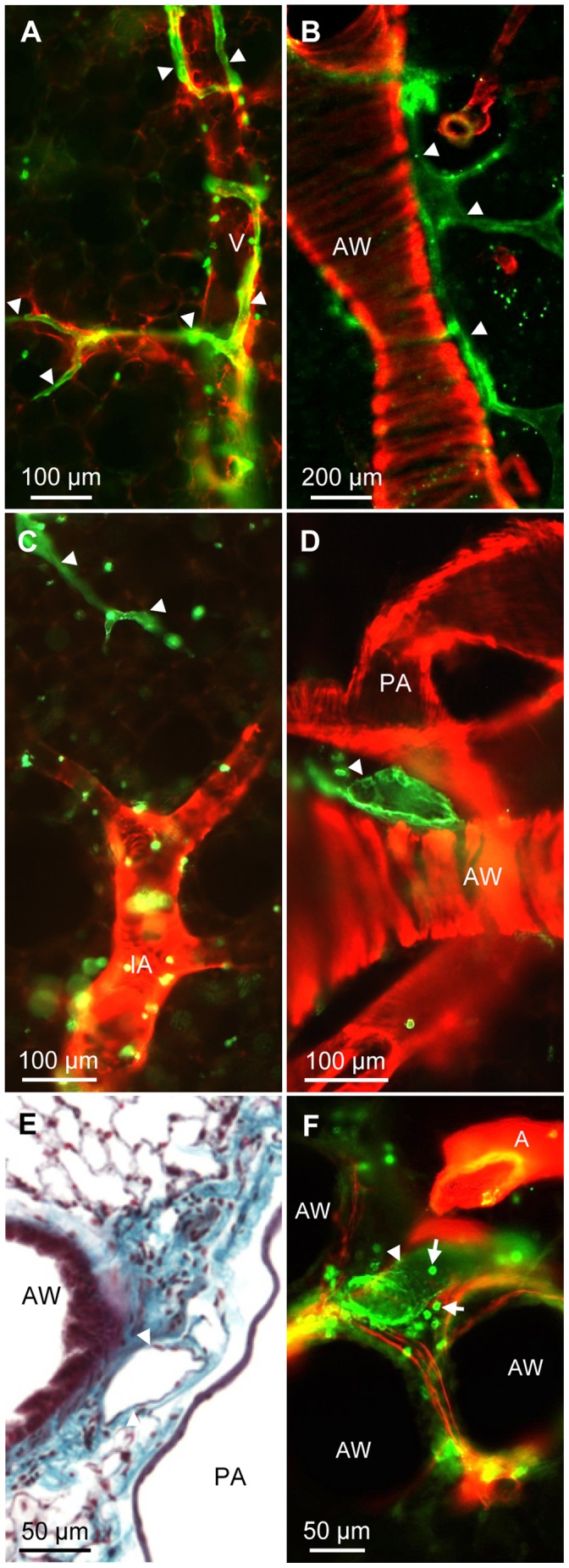
Distribution of lymph vessels in the murine lung with respect to blood vessels and airways. A–D and F) Double-labeling of precision cut lung slices with anti-CD90/Thy-1 antibody (green) and anti-α-smooth muscle actin antibody (red). A) CD90/Thy-1-immunoreactive lymph vessels are found around veins (V) and B) around muscularized airways (AW). C) Intraacinar arteries (IA) are not accompanied by lymph vessels. D, E) Lymph vessels are found frequently in the connective tissue between pulmonary arteries and airways. E) Paraffin section of murine lung stained with Masson Goldner stain. F) Frequently, accumulations of CD90/Thy-1-immunoreactive cells with lymphocyte morphology (arrows) are found around lymph vessels. Arrowheads = lymph vessels.

**Figure 7:**
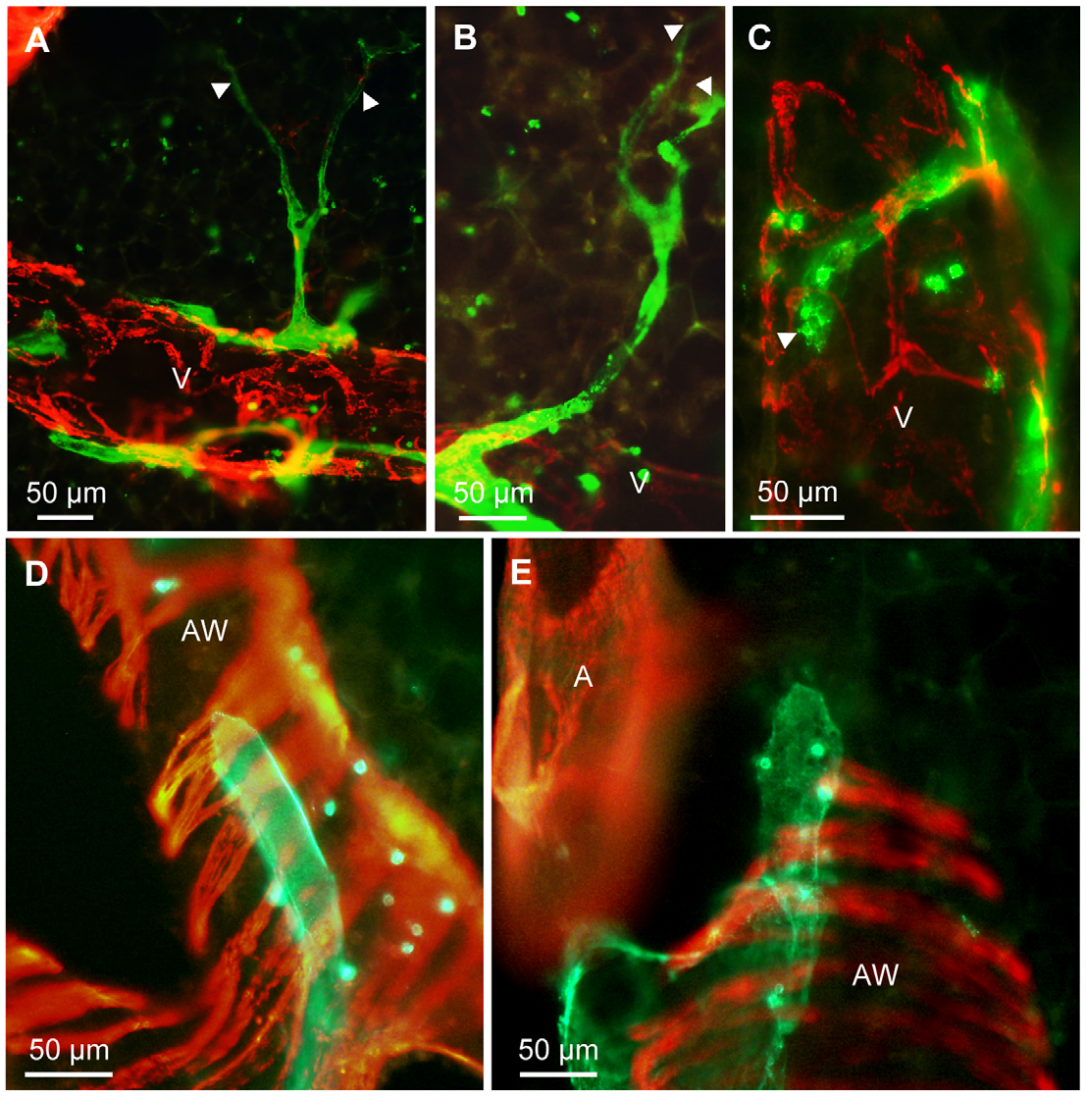
Two separate routes for lymphatics can be identified in murine lungs. Double-labeling of precision cut lung slices with anti-CD90/Thy-1 antibody (green) and anti-α-smooth muscle actin antibody (red). A–C) Lymphatic capillaries (arrowheads) that follow veins (V) begin either in the parenchyma (A, B) or directly on veins (C). D, E) Lymphatic capillaries that follow airways (AW) either begin directly on airways (D) or (E) in the connective tissue between arteries (labeled A in E) and airways.

We did not see qualitative differences in the distribution of lymph vessels in C56BL/6 and Balb/c mice. By staining precision cut lung slices with an antibody against α-smooth muscle actin it is possible to identify arteries, veins and airways by their respective specific smooth muscle cell morphology and distribution (Figure 5 and [14]). By combining staining for CD90/Thy-1 and for α-smooth muscle actin, we found that lymph vessels in the murine lung were either associated with veins or with airways (Figure 6A, B). Intraacinar arteries that are running between alveolar ducts were devoid of lymph vessels (Figure 6C). Lymph vessels were close to pulmonary arteries as they were frequently located in the connective tissue between airways and pulmonary arteries (Figure 6D). Typically this area contained loose connective tissue (Figure 6E) and lymph vessels that were frequently surrounded by CD90/Thy-1-immunoreactive cells with lymphocyte morphology (Figure 6F). Lymph capillaries that subsequently ran with veins either started in the alveolar region (Figure 7A, B) or directly at veins (Figure 7C). Capillaries that ran with airways were starting either directly adjacent at the airway or within the connective tissue between airways and arteries (Figure 7D, E). The analysis is based on n = 9 mice.

### Other CD90/Thy-1-immunoreactive Cells in the Lung

**Figure 8:**
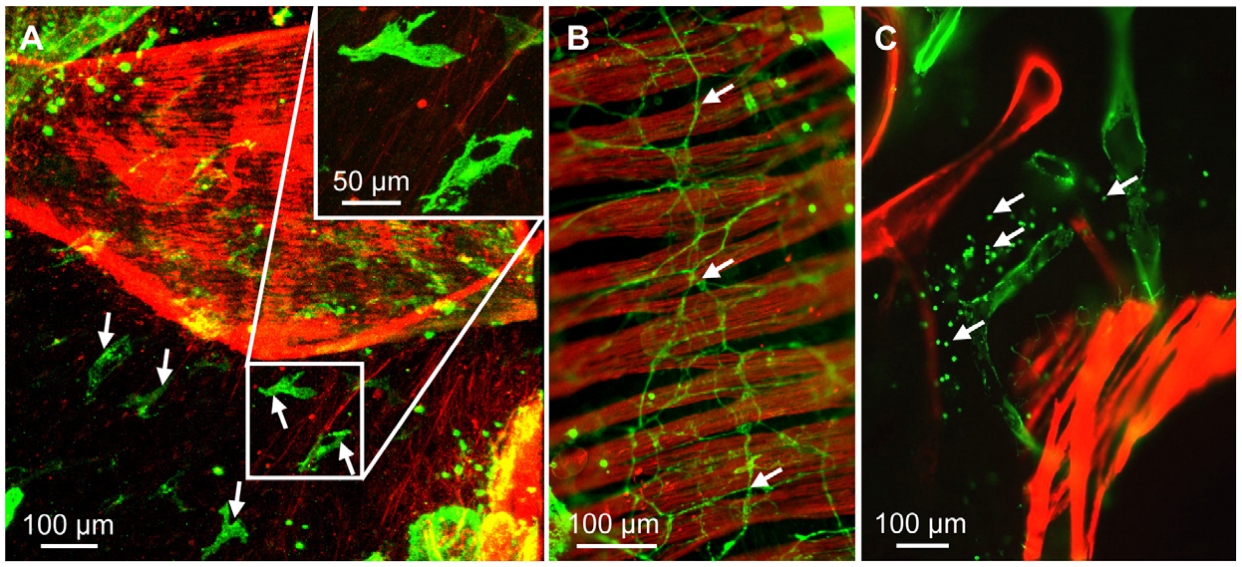
Other cell types are CD90/Thy-1-immunoreactive. Double-labeling of precision cut lung slices with anti-CD90/Thy-1 antibody (green) and anti-α-smooth muscle actin antibody (red). A–C) In addition to lymph vessels, cells with fibroblast morphology (A, arrows) and boxed area in A, nerve fibers (B, arrows) and cells with lymphocyte morphology (C, arrows) exhibit CD90/Thy-1-immunoreactivity.

**Figure 9:**
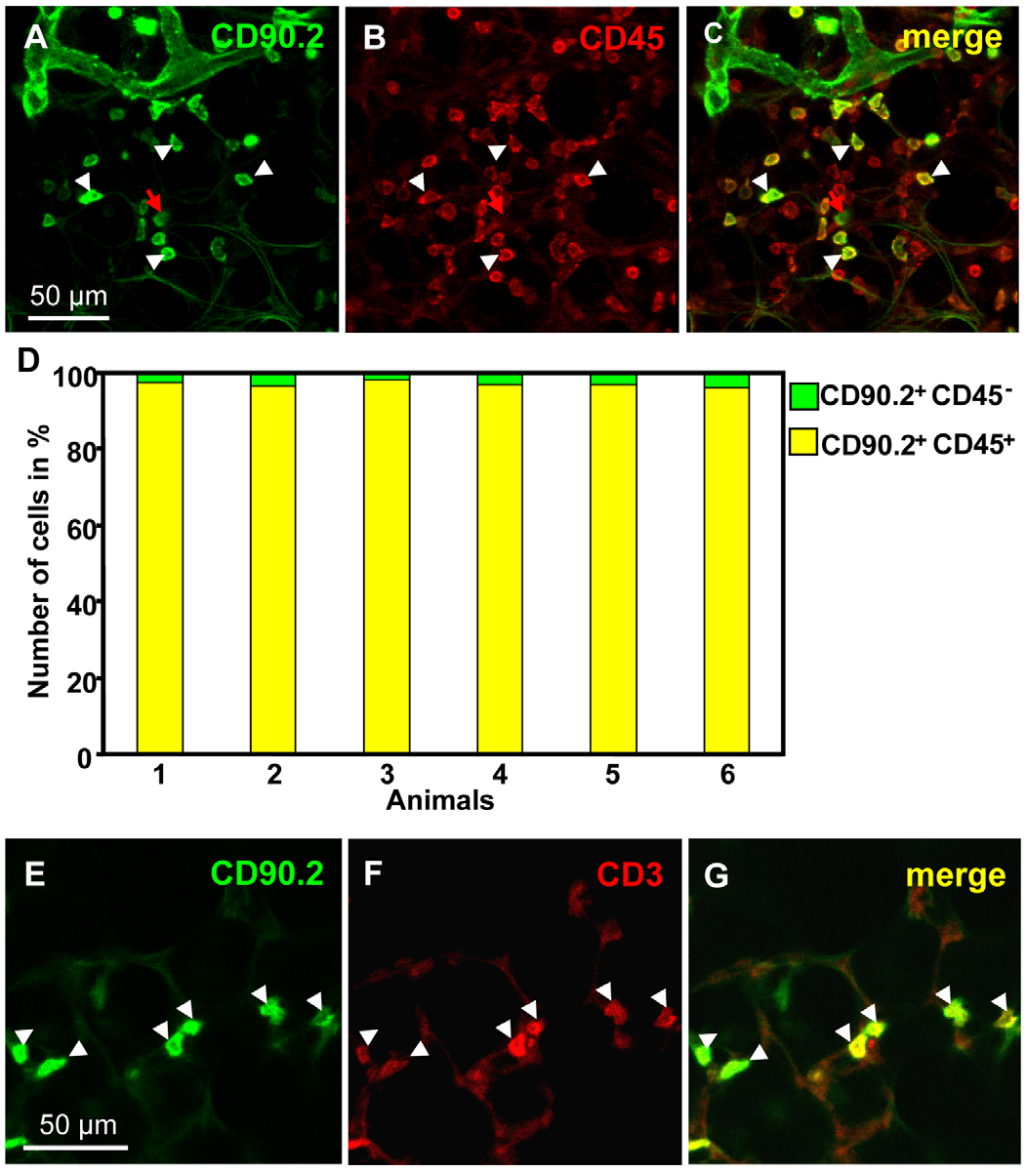
Characterization of CD90/Thy-1-immunoreactive cells with lymphocyte morphology. A–C) Double-labeling of a precision cut lung slices with antibodies against CD90/Thy-1 (green) and CD45 (red), and subsequent quantitative analysis (D) shows that more than 96% of CD90/Thy-1-immunoreactive cells with lymphocyte morphology are also stained with CD45 (white arrowheads, cells positive for CD90/Thy-1and CD45; red arrow, cell immunoreactive for CD90/Thy-1 only). In each animal, 90 to 150 CD90/Thy-1-immunoreactive cells were analyzed. E–G). Double-labeling with antibodies against CD90/Thy-1 (green) and CD3 (red) indicates that most CD90/Thy-1-labeled cells with lymphocyte morphology are also CD3-immunoreactive.

In addition to lymph vessels, other cells in the lung exhibited CD90/Thy-1-immunoreactivity, but were easily distinguishable from lymph vessels in precision cut lung slices. Confined to the immediate hilar region, immunoreactive cells with the morphology of fibroblasts were found (Figure 8A). Around airways, a dense network of nerve fibers was CD90/Thy-1 positive (Figure 8B) and CD90/Thy-1-immunoreactive cells with the morphology of lymphocytes were also observed (Figure 8C). We verified that nearly all of CD90/Thy-1 positive cells are leukocytes by co-staining with an antibody to CD45 and detected co-staining for CD90/Thy-1 and CD45 in 96.92±0.12 (S.E.M., n = 6 animals) (Figure 9A–D). Double-labeling of CD90/Thy-1 with CD3 further indicated that most of the CD90/Thy-1-immunoreactive cells with lymphocyte morphology are indeed T cells (Figure 9E–G, n = 3 animals).

### Detection of Lymph Vessels in Living Tissue

**Figure 10:**
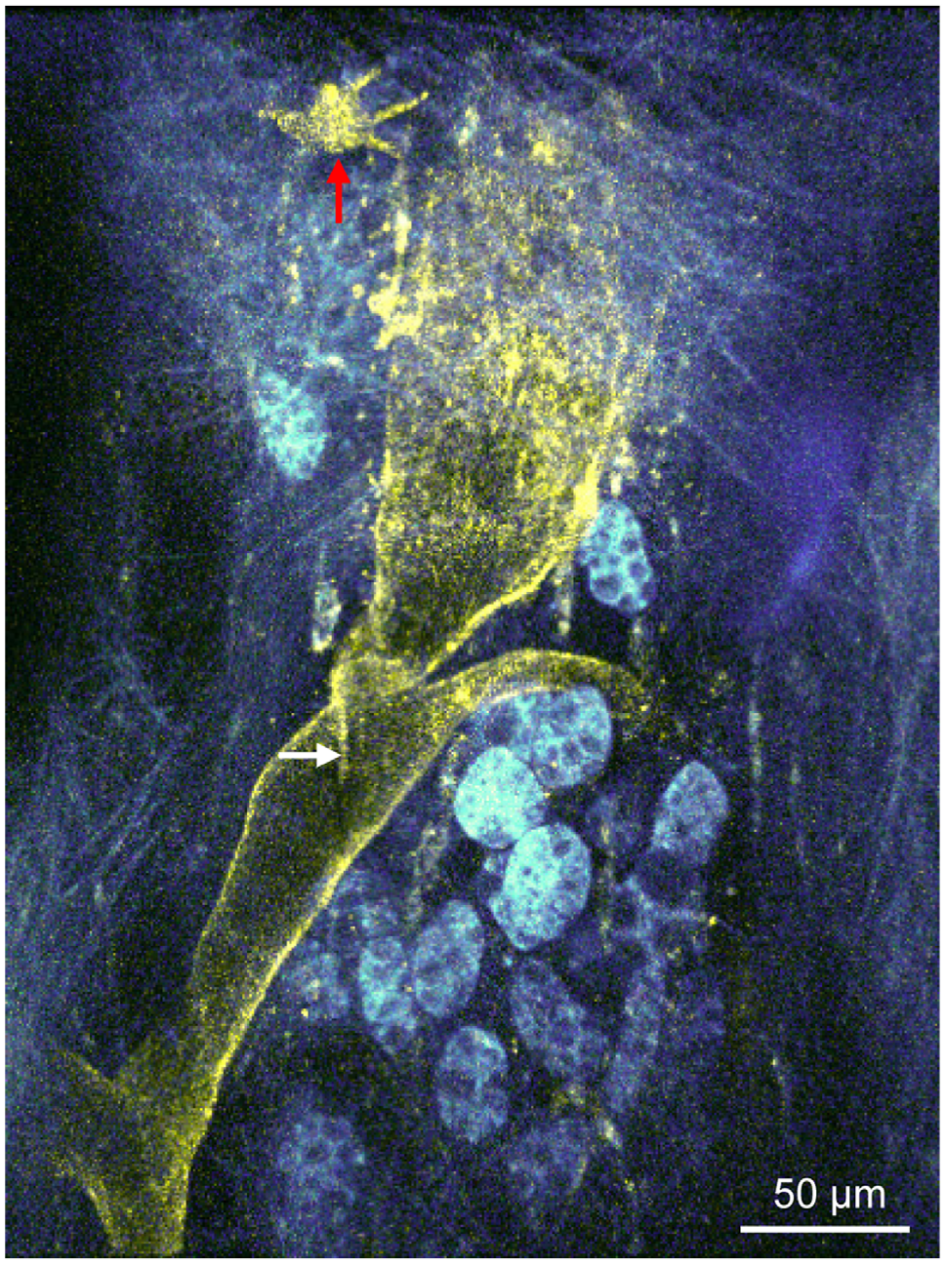
Identification of lymph vessels in the living trachea by *ex vivo* staining with an anti-CD90/Thy-1 antibody. Projection of a z-stack in a living murine trachea *ex vivo* recorded by multiphoton microscopy. Preincubation with an anti-CD90/Thy-1 antibody coupled to FITC stains a lymph vessel (white arrow = lymph vessel valve) and a cell with fibroblast morphology (red arrow) in a living trachea *ex vivo*. Other structures of the tissue are visualized by detection of tissue autofluorescence.

Incubation explanted living murine tracheae with FITC-labeled anti-CD90/Thy-1 antibody stained lymph vessels that could be detected by multiphoton microscopy (Figure 10, and Movie S1). Lymph vessels do not exhibit strong autofluorescence signal in multiphoton microscopy and appear as dark holes. The analysis is based on n = 4 animals.

### Distribution of Lymph Vessels and Lymphocytes after House Dust Mite Challenge

**Figure 11:**
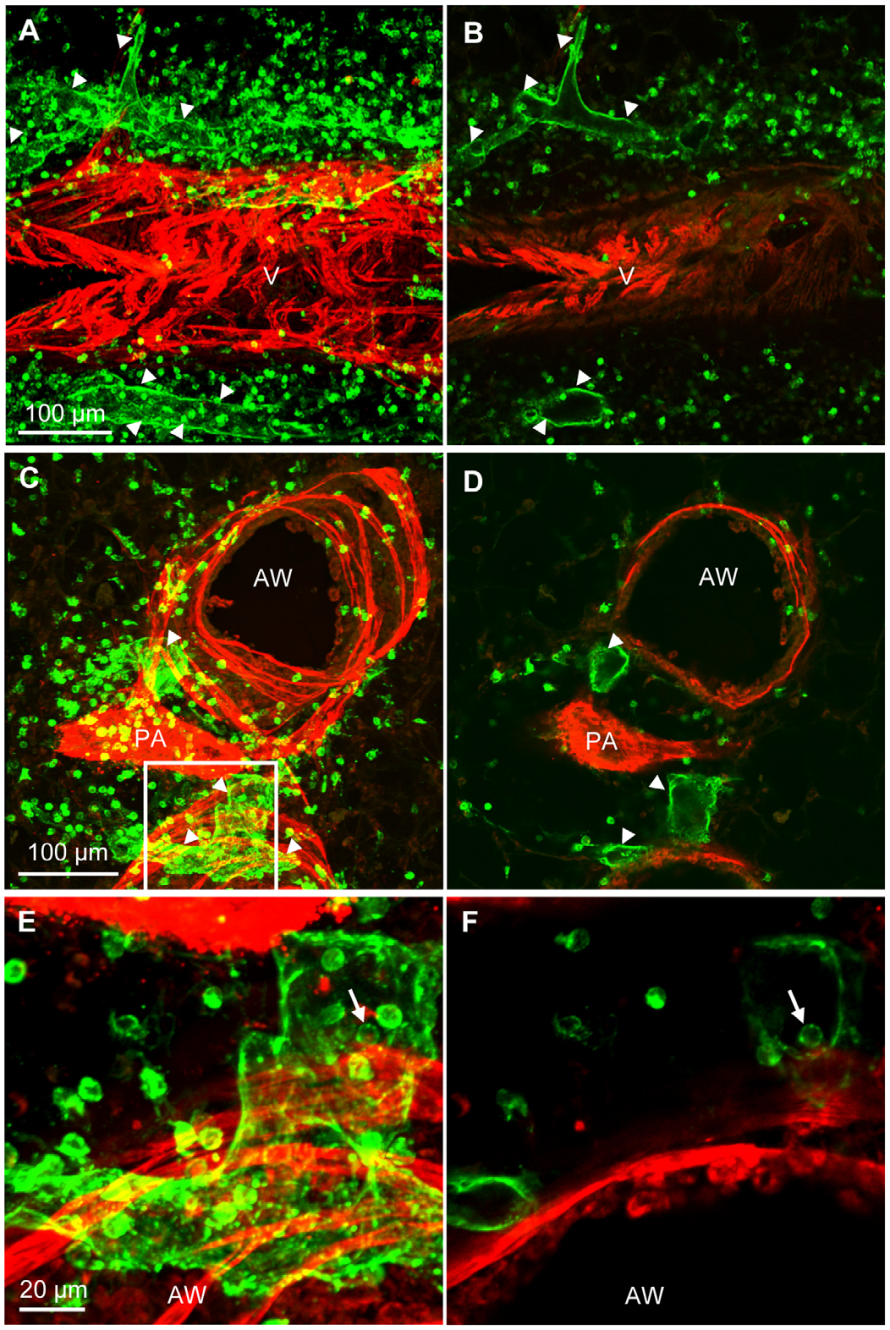
Identification of lymph vessels in murine lungs after house dust mite sensitization and challenge. Precision cut lung slices from house dust mite sensitized and stimulated mice stained with antibodies against CD90/Thy-1 (green) and α-smooth muscle actin (red). A–F) Maximum intensity projections of z-stacks of confocal sections (A, C, E) and single confocal sections (B, D, F) from the z-stacks used to generate projections in A, C, E. While projections (A, C, E) show many T cells that are located around lymph vessels (arrowheads) and can mask them, a single confocal section (B, D, F) allow the unambiguous identification of lymph vessels (arrowheads) and the localization of T cells around and within (arrow in F) lymph vessels. V = vein, AW = airway, PA = pulmonary artery.

**Figure 12:**
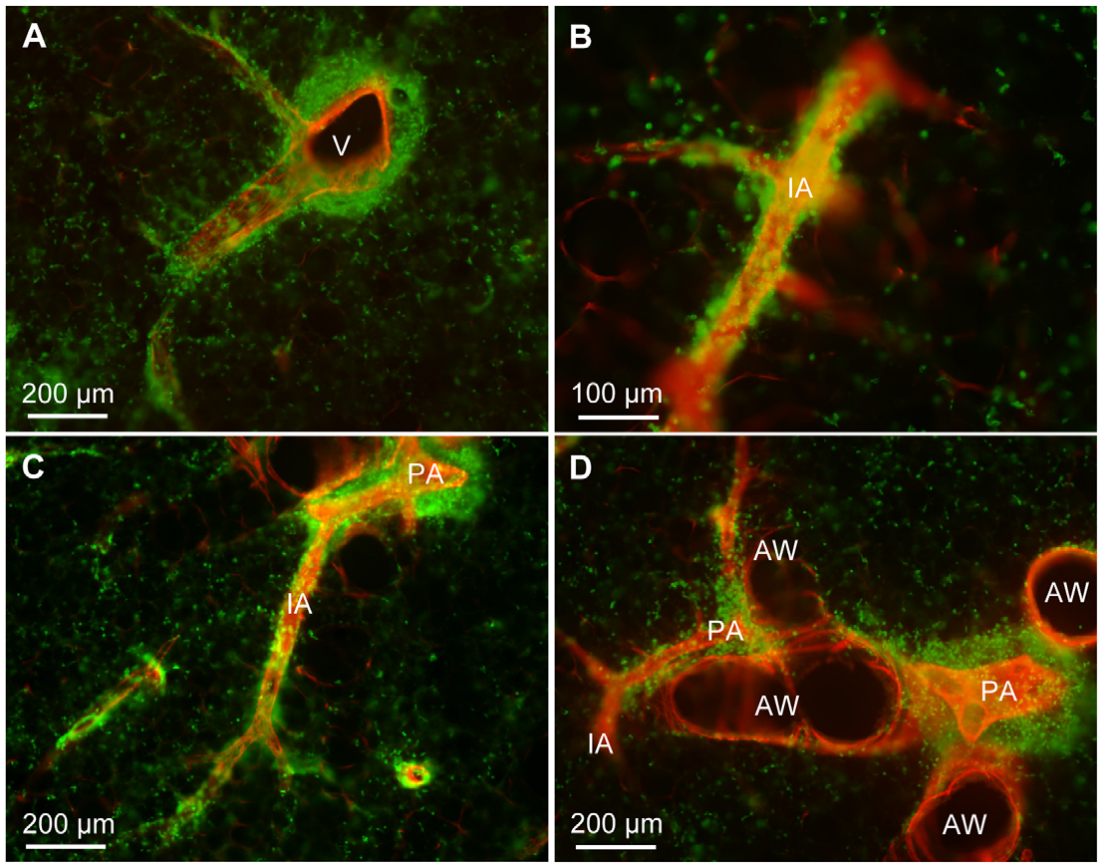
T cell distribution in the murine lung after house dust mite sensitization and challenge. A, B) CD90/Thy-1-immunoreactive T cells are found (A) around veins (V) and (B) around intraacinar arteries (IA). C) Accumulations of T cells around arteries are continuous from intraacinar arteries to pulmonary arteries (PA). D) Around airways (AW), T cells preferentially accumulate around pulmonary arteries.

Exposure to house dust mite extract induced no obvious changes in the distribution and morphology of lymph vessels compared to the PBS treated animals. In both groups CD90/Thy-1 immunostaining was restricted to the lymph endothelium, T cells, fibroblasts and nerve fibers. Although lymph vessels were surrounded by CD90/Thy-1-immunoreactive cells, confocal imaging allowed identification of stained lymph vessels (Figure 11). As T cells were CD90/Thy-1-immunoreactive, we were able to evaluate specifically the distribution of T cells with respect to lymph vessels. Very often, T cells were associated with or even located inside lymph vessels (Figure 11E, F). Frequently, CD90/Thy-1-immunoreactive T cells were found in the alveolar region but accumulated predominantly around veins and arteries. Around veins, T cells were close to lymphatics (Figure 12A). Intraacinar arteries were devoid of lymph vessels but exhibited accumulation of T cells (Figure 12B). This T cell coat often continuously accompanied arteries and was still present when the intraacinar arteries connected the pulmonary arteries that accompanied airways (Figure 12C). In this region T cells were mostly found around arteries and in the connective tissue between arteries and airways that also harbored the lymph vessels (Figure 12D). Around the parts of the airways that were not oriented towards the arteries, markedly fewer lymphocytes were found (Figure 12D). The analysis is based on n = 6 house dust mite treated and n = 6 PBS treated animals.

## Discussion

In this study, we have shown that pulmonary lymph vessels express CD90/Thy-1 and that immunostaining against CD90/Thy-1 can identify lymph vessels under normal and inflammatory conditions. Using CD90/Thy-1 staining in precision cut lung slices, we were able to examine the distribution of lymph vessels as well as T cells in the murine lung under normal and inflammatory conditions.

### Usefulness of CD90/Thy-1 as Marker for Lymph Vessels

Classical markers for lymph endothelium such as podoplanin and LYVE-1 that reliably stain lymph vessels in other organs are not very useful to identify lymph vessels in the lung as staining of other cell types hampers the identification of lymph vessels (c.f. Figure 3). This is due to strong expression in other cell types such as blood endothelial cells and type II pneumocytes [8]. By using precision cut lung slices we were able to show that the anti-CD90/Thy-1 antibody used strongly labeled all sizes of lymph vessels in the lung starting from the initial lymph capillaries to the large vessels that exited the lung at the hilum. As with all other “specific” markers for lymph endothelium, CD90/Thy-1 is not exclusively expressed on lymph endothelium. In the lung it is also found on nerve fibers, fibroblasts and T cells. In principle, the staining of other cell types can be a problem to identify lymphatic vessels and have hampered the use of established markers in lung research [8]. However, nerve fibers, fibroblasts and T cells can be easily and unambiguously distinguished from lymphatics in 200 µm thick precision cut lung slices by their typical morphology. The additional staining of T cells can even be beneficial as this gives an insight into the distribution of T cells with respect to lymphatics without the need for multiple antibodies. Although it is reported that CD90/Thy-1 is expressed on human endothelial cells [16], we did not observe any CD90/Thy-1-immunoreactivity on blood vessels in healthy animals or in the model of experimental airway inflammation used. From our data we conclude that anti-CD90/Thy-1 antibodies can be used to examine the morphology as well as the distribution of the lymphatic system in the murine lung. Since we could also strongly label lymph vessels in the freshly excised living mouse trachea without any permeabilization step, CD90/Thy-1 is accessible for antibodies on the surface of lymph vessels. It is therefore feasible to use the antibody to detect lymph vessels in *in vivo* and *ex vivo* imaging experiments and should also be suitable for FACS analysis of lymphatic endothelial cells.

### Anatomy of the Murine Lung Lymphatics and Possible Routes for Extracellular Fluid Drainage

**Figure 13:**
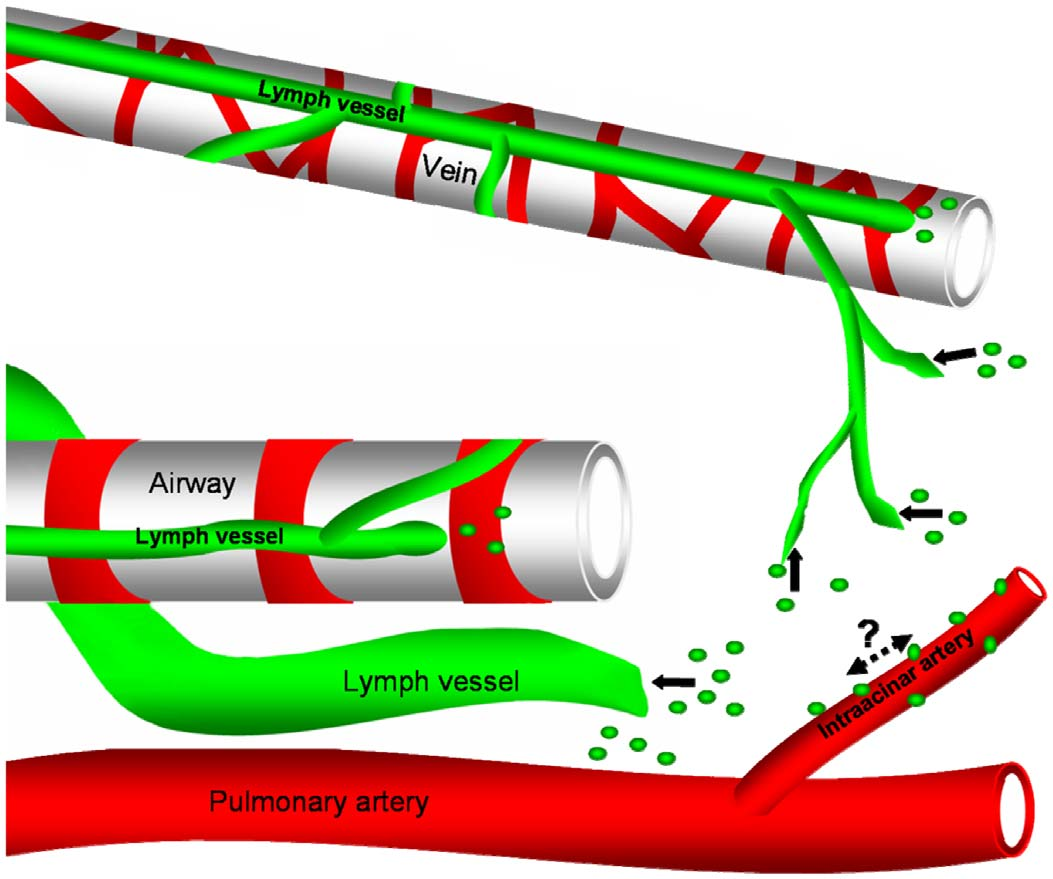
Scheme of lymph vessels and model of cell exit from the murine lung tissue. Two separate routes for lymph vessels exist. One begins in the parenchyma and leaves the lung via veins and the other begins around airways or in the connective tissue between airways and arteries and follows the airways to leave the lung. Cells that have left the vasculature in the alveolar region can enter the lymphatic system by migrating to lymph vessels around veins or by migration to lymph vessels around airways, possibly by using intraacinar arteries as guide.

By using 200 µm thick precision cut lung slices and staining against α-smooth muscle actin we have previously shown that arteries, veins, and airways can be identified by their specific distribution of smooth muscle cells [14]. Co-staining with an anti-α-smooth muscle actin antibody allowed us to define the distribution of the lymphatic vasculature of the lung with respect to blood vessels and airways. We were able to identify two different lymphatic drainage routes. One begins in the parenchyma and leaves the lung via the veins. The other begins around the airways and in the connective tissue between arteries and airways and leaves the lung via the airways. In contrast to the human lung, we did not observe a separate network in the pleura indicating that pleural drainage occurs primarily via the lymphatic system around the veins. Based on the distribution of both systems, most of the extracellular fluid of the alveolar area will be drained via the vein associated lymphatics, whereas extracellular fluid that is generated around the airways will primarily be drained via airway associated lymph vessels. Intrapulmonary lymphatics were devoid of smooth muscle cells with the exception of large lymph vessels at the lung hilum. This indicates that the intrapulmonary transport of lymph in both systems primarily relies on compression of lymph vessels during breathing and not on active contraction of smooth muscle cells. The presence of valves within lymph vessels supports this idea. Our results on the anatomy of murine lung lymphatics derived from staining of precision cut lung slices by anti-CD90/Thy-1 antibody staining are summarized in Figure 13.

### Possible Routes for Immune Cells to Leave the Lung

Following application of house dust mite extract, we found the expected accumulation of T cells adjacent to airways but also in the alveolar region. At first sight it is surprising that the effects of house dust mite extract application were not confined to airways. However, the applied house dust mite extract could also reach the alveolar space. In fact, the alveolar region seems to be an important area for antigen uptake as Thornton and coworkers have recently shown. Using an OVA model of allergic airway inflammation, the authors demonstrated that most of the OVA was taken up by dendritic cells in the alveolar region and not in the airways [17]. Thus, the increased amount of T cells, we observed indicates that house dust mite reached the alveolar space and initiated an immune response there. Based on the lymph vessel distribution, we expected that immune cells in the alveolar region will travel to draining lymph nodes via lymphatics around veins. Indeed, we found accumulations of CD90/Thy-1-immunoreactive cells around veins often close to lymphatics. Furthermore, we detected also CD90/Thy-1-immunoreactive T cells in lymph vessels, indicating that indeed, T cells entered lymph vessels and are used for egress. However, we also found substantial accumulation of T cells around intraacinar arteries despite the fact that these vessels were consistently devoid of lymph vessels. We also observed that this lymphocyte cuff around arteries extended up to pulmonary arteries that accompanied the airways. From our experiments we cannot infer how T cells reach intraacinar arteries and where they migrate. However, it should be noted that the periarterial space has been recognized as a specific area for immune reactions in a variety of lung diseases [18], [19]. A possible explanation is that there is a specialized capillary network that accompanies arteries which allows cell recruitment of inflammatory cells [19]. However, it has been recently described that dendritic cells that take up antigen in the alveolar region, migrate to airway adjacent areas [17]. It is therefore tempting to speculate that cells from the alveolar region use intraacinar arteries as guides to reach the airways.

### Possible Functions of CD90/Thy-1 on Lung Lymphatics

The function of CD90/Thy-1 on lymphatic endothelial cells has not been directly analyzed *in vivo*. The only existing publication that addressed the expression of CD90/Thy-1 in lymphatic endothelium demonstrated that blockade of CD90/Thy-1 impaired the attachment of tumor cells to lymphatic endothelium in mouse and prevented attachment of mononuclear and polymorph nuclear leukocytes to cultured human lymphatic endothelium [9]. This is in line with the proposed role for CD90/Thy-1 as a mediator of leukocyte adhesion to endothelial cells [20], [21], [22]. To our knowledge, no other publication has yet specifically addressed the function of CD90/Thy-1 on lymphatic endothelium. However, indirect evidence points to a possible involvement of CD90/Thy-1 on lymphatic endothelium in pulmonary inflammatory processes as CD90/Thy-1 deficient mice show a reduced number and altered composition of leukocytes during inflammation [20]. The authors did not address the involvement of CD90/Thy-1 expression on lymphatic endothelium and interpreted their results as a recruitment problem for inflammatory cells to the airways. Alternatively, it is possible that altered function of lymph endothelium contributes to the phenotype since the effects observed were not due to the expression of CD90/Thy-1 on T cells as transfer of wild-type bone marrow from CD90/Thy-1-deficient mice restored T cells that express CD90/Thy-1 but did not restore the original composition and number of leukocytes during inflammation.

### CD90/Thy-1 on Pulmonary Fibroblasts

CD90/Thy-1 has been shown to be present on human, rat and mouse lung fibroblasts and is down-regulated or absent in fibroblasts of fibrotic lesions of patients with idiopathic lung fibrosis [12], [23], [24]. Our observations confirm the presence of a CD90/Thy-1 positive fibroblast population which is localized close to the lung hilum. However, we did not find CD90/Thy-1 positive fibroblasts in other areas of the lung. It is therefore possible that the increased fibrosis detected in CD90/Thy-1 knockout mice after bleomycin application [23] could also be attributed to changes in lymph vessel function.

## Conclusions

In conclusion, our data shows that CD90/Thy-1 is expressed on lymph endothelium in the murine lung and can be used as a reliable marker to examine the intrapulmonary lymphatic system when used in combination with precision cut lung slices. This technique opens up the possibility to get further insight into the function of the intrapulmonary lymphatic system. Since very well validated monoclonal antibodies against CD90/Thy-1 are commercially available, a reliable source for the reagents exists and researchers do not have to rely on polyclonal antibodies on the market that can change in their quality over time and have to be validated for their specificity after obtaining a new batch.

## Supporting Information

Movie S1 **Identification of lymph vessels in the living trachea by** ***ex vivo*** **staining with an anti-CD90/Thy-1 antibody.** Sections of the z-stack projection shown in figure 10 recorded by multiphoton microscopy. Preincubation of a living murine trachea *ex vivo* with an anti-CD90/Thy-1 antibody coupled to FITC stains a lymph vessel and a cell with fibroblast morphology. Other structures of the tissue are visualized by detection of tissue autofluorescence.
(AVI)
